# The British Nurses' Association

**Published:** 1888-03-24

**Authors:** 


### THE BRITISH NURSES ASSOCIATION.

You have asked for the opinion of your readers about the British Nurses Association, and, speaking for other besides myself, I can safely say that we strongly object to a form of registration which will put *any* nurse who has worked three years on a footing with a thoroughly-trained nurse. As the scheme stands, a "Sairey Gamp" who has acted in her village for three years can demand such support as the society can give her. The whole theory of one central register for nurses is a mistake; the certificates given by hospitals are always a good guarantee, and ought not to be set aside for those given by a body which can have no complete or proper knowledge of those to whom they grant them. If only the many doctors who have lent their name to the British Nurses Association could see where the scheme tends, they would surely proceed with more caution. Some of the many antagonistic societies now being started amongst nurses must infallibly fall to the ground, and your warning to nurses to join no such societies in a hurry is heartily seconded by many. F. H. M.

The London Hospital, March 13.

